# Validation of the Arabic Version of the Kessler Psychological Distress Scale (K6) among college students in Kuwait

**DOI:** 10.1192/j.eurpsy.2023.2054

**Published:** 2023-07-19

**Authors:** B. M. Alansari

**Affiliations:** psychology Department, Kuwait University, Kaifan, Kuwait

## Abstract

**Introduction:**

The Kessler Psychological Distress Scale (K6) is a psychometrically robust measure of psychological distress for adult populations, the most commonly used screening measure in the general population. It is used in epidemiological surveys globally, including the World Health Organization and World Mental Health Survey. Recently, there has been a research interest in examining the psychometric properties of the K6 in college students’ populations.

**Objectives:**

To examine the reliability, validity and factor structure of the Arabic adaptation K6 in Kuwaiti college students.

**Methods:**

The participants were 1402 individuals (509 males, 893 females) Kuwait University undergraduates, aged 18–35 years-old mean age = 21.50 ± 4.87. The Arabic versions of The Kessler Psychological Distress Scale (K6), The Depression Anxiety Stress Scales DASS-42, Beck Depression Inventory-II, and Beck Anxiety Inventory –BAI were administered to participants including demographics. The internal consistency reliability, factor structure, and convergent validity of the (K6) with DASS-42, BDI-II, & BAI were computed.

**Results:**

Internal consistency was satisfactory for the K6 (Cronbach’s alpha =0.80 for males & 0.79 for females). The results revealed significant gender differences in distress with a favor for females (*f*=8.95, *p.* >.003). Principal component analyses (PCA) showed that a K6 one -component solution explains %61.51 of the total variance for males and %57.23 for females. The k6 correlates with DASS Stress (r=.76), DASS Depression (r=.57), DASS Anxiety (r=0.76), BAI (r=.73) and BDI-II (r=46).

**Conclusions:**

The K6 provides satisfactory validation, and can be recommended as a measure of distress among Arab college students.

**Disclosure of Interest:**

None Declared

